# Limited gains in native parasitoid performance on an invasive host beyond three generations of selection

**DOI:** 10.1111/eva.13504

**Published:** 2022-11-02

**Authors:** Shelley Linder, Benjamin J. M. Jarrett, Philip Fanning, Rufus Isaacs, Marianna Szűcs

**Affiliations:** ^1^ Department of Entomology Michigan State University East Lansing Michigan USA; ^2^ Department of Biology Lund University Lund Sweden; ^3^ School of Biology and Ecology University of Maine Orono Maine USA

**Keywords:** ectoparasitoid, endoparasitoid, evolutionary trade‐offs, parasitoid host range evolution, spotted wing drosophila

## Abstract

Co‐evolved natural enemies provide sustainable and long‐term control of numerous invasive insect pests, but the introduction of such enemies has declined sharply due to increasing regulations. In the absence of co‐evolved natural enemies, native species may attack exotic invasive pests; however, they usually lack adaptations to control novel hosts effectively. We investigated the potential of two native pupal parasitoids, *Pachycrepoideus vindemmiae* and *Trichopria drosophilae*, to increase their developmental success on the invasive *Drosophila suzukii*. Replicated populations of the two parasitoids were subjected to 10 generations of laboratory selection on *D. suzukii* with *Drosophila melanogaster* serving as the co‐evolved host. We assessed developmental success of selected and control lines in generations 0, 3, and 10. Changes in host preference, sex ratio, development time, and body size were measured to evaluate correlated responses with adaptation. Both parasitoid species responded rapidly to selection by significantly increasing their developmental success on the novel host within three generations, which remained constant for seven additional generations without further improvement. The generalist parasitoid species *P. vindemmiae* was able to reach similar developmental success as the control populations, while the performance of the more specialized parasitoid *T. drosophilae* remained lower on the novel than on the co‐evolved host. There was no increase in preference towards the novel host over 10 generations of selection nor were there changes in development time or body size associated with adaptation in either parasitoid species. The sex ratio became less female‐biased for both parasitoids after three generations of selection but rebounded in *P. vindemmiae* by generation 10. These results suggest that a few generations of selection may be sufficient to improve the performance of native parasitoids on invasive hosts, but with limits to the degree of improvement for managing invasive pests when exotic co‐evolved natural enemies are not available.

## 1 INTRODUCTION

Invasive insect pests are most effectively controlled in the long term by co‐evolved natural enemies. These may be introduced as part of classical biological control programs or can be introduced accidentally along with the pest (Abram et al., 2022; Heimpel & Cock, 2018). However, new classical biological control introductions have declined significantly due to increasing regulations and costs associated with foreign exploration for natural enemies and their safety testing (Van Driesche et al., 2020). At the same time, exotic insect introductions keep rising (Huang et al., 2011), and in the absence of effective natural enemies, their populations can increase unchecked. Native natural enemies, including insect parasitoids, could potentially provide control of exotic pests in the absence of classical biological control agents. Initially, however, native parasitoids tend to be inefficient at attacking invaders due to the novelty of the exotic host (Abram et al., 2017; Chabert et al., 2012; Costi et al., 2020; Konopka et al., 2018; Kruitwagen et al., 2018). Over time, the richness and efficacy of native parasitoid complexes can increase, sometimes reaching similar levels of parasitism as on native hosts within decades or centuries (Cornell & Hawkins, 1993; Grabenweger et al., 2010; Gröbler & Lewis, 2008; Vindstad et al., 2013). Native parasitoids on novel hosts may accumulate relatively quickly, especially if there are native species in the invaded range that are ecologically and taxonomically similar to the invaders (Cornell & Hawkins, 1993). However, in the wild, adaptation of a parasitoid to novel hosts can be slow as it may require physiological and behavioral changes to take place without strong directional selection given that parasitoids have a choice between the ancestral native and novel exotic hosts (Kawecki & Ebert, 2004; Strauss et al., 2006). In addition, environmental variability and phenological mismatch between the novel hosts and resident parasitoids can also interfere with adaptation (Bonsignore & Bernardo, 2018).

Laboratory selection experiments have revealed that under strong selection, parasitoid host range can evolve within a few generations since host use is genetically determined and there is substantial additive genetic variation in the ability of populations to parasitize various host species (Carton, 1991; Dion et al., 2011; Henry et al., 2008; Henter, 1995; Hufbauer, 2002; Kraaijeveld et al., 1998). In biological control programs, laboratory selection has been used in rare cases to increase the performance of parasitoids on sub‐optimal hosts (Lirakis & Magalhães, 2019) and to improve effectiveness of native parasitoids at attacking invasive hosts (Jarrett et al., 2022; Kruitwagen et al., 2021). Laboratory selection has also been used to assess the possibility of postintroduction evolution of exotic parasitoids to infest native nontarget hosts more effectively (Hopper et al., 2021). The few studies to date highlight the variable responses of parasitoids to selection imposed by a novel host that may stem from differences in the parasitoid's initial host range, life history traits, or standing genetic variation within the population. For example, out of three native parasitoids that were selected for improved performance on the invasive spotted wing drosophila (*Drosophila suzukii*) (Matsumura) (Diptera: Drosophilidae), two have been shown to increase developmental success on the invasive host (Jarrett et al., 2022; Kruitwagen et al., 2021). Selection somewhat increased the attack rate but not the developmental success of the larval parasitoid *Leptopilina heterotoma* (Thomson) (Hymenoptera: Figitidae) (Kruitwagen et al., 2021), while developmental success of two pupal parasitoids *Pachycrepoideus vindemmiae* (Rondani) (Hymenoptera: Pteromalidae) and *Trichopria drosophilae* (Perkins) (Hymenoptera: Diapriidae) increased significantly after only three generations of selection (Jarrett et al., 2022). These recent studies provide a foundation for future applications in the field of biological control, where the performance of native natural enemies may be improved in the laboratory before release to combat invasive pests (Jarrett et al., 2022; Kruitwagen et al., 2018). However, for this approach to become viable, we need a deeper understanding of how quickly adaptation may happen, what the limits of adaptation may be, and how correlated responses to adaptation may influence the effectiveness of laboratory‐improved parasitoids.

We know from multiple systems including Drosophilids (Jarrett et al., 2022), various aphid (Dion et al., 2011; Henry et al., 2010; Hopper et al., 2021), and moth (Jones et al., 2015) host species that in the laboratory parasitoids can evolve increased developmental success on a novel host rapidly, often within 3–10 generations. It is unclear how universal these responses may be among different parasitoid species and if there may be an ideal length for laboratory selection that balances increased effectiveness and the cost of conducting long selection experiments. Some studies indicate that response to selection can be as rapid as 2–4 generations, beyond which further improvement of parasitoids is negligible (Dion et al., 2011; Hopper et al., 2021). However, there are also examples of continued improvement of parasitoid performance on a sub‐optimal host for up to 40 generations of selection (Henry et al., 2008).

Adaptation of parasitoids to novel hosts can come with correlated responses that may range from negative to positive. Negative trade‐offs were shown for *Aphidius ervi* (Haliday) (Hymenoptera: Braconidae), where lines selected for increased virulence on pea aphids harboring the symbiont *Hamiltonella defensa* (Pseudomonadota: Enterobacteriaceae) had smaller size (Dion et al., 2011) and adaptation of *Venturia canescens* (Gravenhorst) (Hymenoptera: Ichneumonidae) to a novel host came at a cost of reduced performance on the original host (Jones et al., 2015). Some correlated responses can help with maintaining adaptations by promoting reduced gene flow between populations adapted to a new host and those using ancestral hosts. For example, *A. ervi* that evolved increased virulence on a new aphid host species showed higher acceptance of the new host over the ancestral host (Henry et al., 2008). Such fitness trade‐offs or changes in host fidelity can be important in determining how experimentally improved parasitoids may perform in the field.

In a previous study, we showed that two parasitoid species, *P. vindemmiae* and *T. drosophilae*, from North America substantially increased development success on the invasive *D. suzukii* just after three generations of selection (Jarrett et al., 2022). Here, we report how developmental success changed with an additional seven generations of selection for both species. In addition, we also assessed host preference of selected and control populations in generations 0, 3, and 10 and evaluated three potential trade‐offs associated with adaptation: sex ratio, development time, and body size.

## 2 MATERIALS AND METHODS

### 2.1 Study system

*Drosophila suzukii* is native to Asia and was introduced to the Americas and Europe in the late 2000s where it has become a major pest of soft fruit crops (Asplen et al., 2015). It is primarily controlled by insecticides in orchards (Lee et al., 2019). Biological control that could suppress *D. suzukii* numbers in noncrop areas has remained under 10% by native parasitoids a decade after introduction (Lee et al., 2019). We used two cosmopolitan pupal parasitoids (*P. vindemmiae* and *T. drosophilae*) that are adapted to develop on *Drosophila melanogaster* but for which *D. suzukii* represents a novel host. These parasitoids show inter‐ and intraspecific variation in their ability to parasitize *D. suzukii* (Chabert et al., 2012; Stacconi et al., 2015). *Pachycrepoideus vindemmiae* has a wide host range, attacking at least 60 Dipteran species in multiple families and genera (Wang & Messing, 2004). Conversely, the host range of *T. drosophilae* is restricted to the family Drosophilidae (Carton et al., 1986). Both species are found worldwide, but our laboratory colonies were initiated from North American populations as described in Jarrett et al. (2022). We used *D. melanogaster* as the co‐evolved “control” host since it has a >200‐year history in North America (David & Capy, 1988). Additionally, we demonstrated that our two parasitoid species had high initial developmental success on *D. melanogaster* (Jarrett et al., 2022).

### 2.2 Parasitoid and fly rearing

Both the fly and parasitoid colonies were maintained at 25 ± 2°C with a 16L:8D photoperiod and 85% relative humidity. *Pachycrepoideus vindemmiae* populations were founded by two wild‐caught females (identified by P. Fanning using Gibson et al., 1997). They were captured in 2018 in Michigan, USA (42.6749, −84.4897). *Trichopria drosophilae* populations were founded by 30 individuals and originated from a laboratory colony in California, USA, which originally had been founded by six females and five males but were later periodically augmented with field‐caught individuals (Wang et al., 2016).

Both parasitoid species were reared on *D. melanogaster* to maintain large populations (*n* = 1800 individuals for each species) prior to experiments. The flies were provided with the Drosophila Species Stock Center cornmeal diet in drosophila vials (2.5 × 9.5 cm; Lab‐Express) with foam stoppers (Genesee Scientific). To rear the fly species, each vial for *D. melanogaster* was initiated with 10 individuals and for *D. suzukii* with 20 individuals. This resulted in similar numbers of pupae available for parasitism given the different fecundities of the fly species (Jarrett et al., 2022).

### 2.3 Experimental evolution experiments

To test how the two parasitoid species would respond to selection on *D. suzukii*, we set up three replicated selection lines for both parasitoid species on *D. suzukii* and three replicated control lines on *D. melanogaster* (Jarrett et al., 2022; Kawecki & Ebert, 2004). The starting populations for each replicated selection and control line had 300 individuals (80% females, mimicking the observed sex ratio after rearing on *D. melanogaster* and after rearing on *D. suzukii* for one generation; Jarrett et al., 2022). By generation 3, the sizes of replicated populations increased to 800–1000 individuals and were capped at this size for the duration of the experiment.

To initiate the replicated populations, parasitoids (*n* = 300 per population) were randomly divided into 12 vials (~25 parasitoids per vial). The adult wasps were provided with a small strip of honey and left to parasitize fly pupae for 48 h. Parasitoids were transferred to fresh vials with fly pupae every 48 h until a minimum of 40 vials contained parasitized pupae. Vials were monitored every other day for both fly and parasitoid emergence for 50 days. Emerging parasitoids were collected and provided with honey until at least 100 individuals had emerged. We then initiated the next discrete generation by providing the appropriate fly hosts to the emerged wasps. We continued to collect emerging parasitoids and provide them with fly pupae until the wasp population reached at least 800 individuals. The first 100 parasitoids and then the parasitoids emerging daily were allowed to mate at random. Once the population reached 1000 individuals, no more parasitoids were added. Rearing continued in this manner for 10 generations.

Individual fitness measurements were taken at three different time points during the experiment: generation 0, 3, and 10. Following generations 3 and 10, 200 randomly chosen individuals were removed from each of the replicated selection and control lines of both parasitoid species and placed in a common garden host environment (*D. melanogaster*). All lines were reared on *D. melanogaster* for one generation following generation 3 and for two generations following generation 10. This was done to control for phenotypic plasticity and maternal effects (Kawecki et al., 2012). Individual females emerging from the common garden were randomly chosen from each replicate population to be used in assays. Before all assays, the female was placed in a vial with honey and at least one conspecific male for 48 h to ensure she was mated. We refer to these assay results as generation 3 and 10 because those numbers reflect the number of generations spent on the selection host even though additional 1–2 generations were spent in the common garden on the control host *D. melanogaster* prior to assays.

### 2.4 Developmental success and trade‐off assays

#### 2.4.1 Generation 0

We first tested the initial developmental success of the two founding parasitoid populations in generation 0 on both *D. suzukii* and *D. melanogaster* to establish baseline developmental success rates prior to selection. For both parasitoid species, initial developmental success rates were assessed by offering individual female pupae of either *D. melanogaster* or *D. suzukii*. This was done for each of the three populations before the parasitoids were separated into control and selection lines.

In all assays, individual mated females from the common garden generations were placed in a 60 × 15 mm Petri dish arena (Thermo Fisher Scientific) with 10 host pupae. The female parasitoid was removed after 48 h and discarded. The Petri dishes were monitored for emergent flies and parasitoids for 5 weeks. Additionally, the sex of the emergent parasitoids was recorded, to measure the offspring sex ratio.

#### 2.4.2 Generation 3

To test for adaptation, the developmental success of both the selection and control lines were assessed on *D. suzukii* and *D. melanogaster*, respectively, following the generation in the common environment (*D. melanogaster*) (Jarrett et al., 2022). We only assayed females on the species on which they evolved to address the main question of whether native parasitoids could adapt to an invasive host. Therefore, there were three replicates of the control line assayed on *D. melanogaster* and three replicates of the selection line assayed on *D. suzukii* using 15–20 individual females per replicate population. Assays were conducted using the same methods described above.

#### 2.4.3 Generation 10

In generation 10, we assayed individual females from the selection and control lines on their respective host using the same methods as described above. In this generation, we also measured the development time for all offspring emerging during assays. Development time for each emerging offspring was defined as the number of days between the removal of the female wasp from the arena and their emergence.

We also saved the individual female parents emerging from the common garden in 90% ethanol at −80°C. These parasitoids were weighed to test for a trade‐off between body size and adaptation to *D. suzukii*. Measurements were taken using a microbalance with a precision of 0.001 mg (Model XP 26, Mettler Toledo).

### 2.5 Host preference

After generations 0, 3, and 10, host preference was assessed by giving 25 randomly chosen individual females a choice between the novel and the ancestral hosts. Randomly chosen females emerging from the common garden were held with a male from the same population for at least 24 h for mating. They were then provided with 10 *D. melanogaster* and 10 *D. suzukii* pupae in a 60 × 15 mm Petri dish arena. The *D. melanogaster* pupae were placed on one side of the arena and the *D. suzukii* pupae were placed on the opposite side. A thin paper towel barrier was placed down the middle of the arena. This allowed the female to cross but prevented the two different host species from mixing. The female was removed after 48 h, and the pupae were separated by host species to monitor the emergence of flies and wasps in these paired samples.

### 2.6 Statistical methods

All analyses were performed in R 3.5.1 (R Core Team, 2013). Generalized linear mixed models and linear mixed models were constructed using the *lme4* package (Bates et al., 2015). All post hoc pairwise comparisons were performed in the *emmeans* package (Lenth et al., 2021).

#### 2.6.1 Developmental success

For each parasitoid species, we compared the developmental success of the selection and control lines on their respective hosts in generations 0, 3, and 10. Two different metrics were used to assess developmental success. First, developmental success was coded as a binomial outcome: “1” if there were any emerging parasitoid adults in an assay trial or “0” if no parasitoids emerged. For the second model, only those replicates that had successful emergence were used. Here, we compared the proportion of parasitoids emerging from the different host species. We refer to this metric as “emergence rate,” which was calculated by dividing the number of adults emerging by the total number of pupae offered, and as such, the data are bounded between 0 and 1.

For both analyses, we used generalized linear mixed models with a binomial distribution. The fixed effects were the generation (0, 3, or 10), treatment (rearing host of *D. suzukii* for the selection lines and *D. melanogaster* for the control lines), and the replicate population nested within treatment (rearing host) was included as a random effect. We tested for an interaction between generation and rearing host using the *anova* function with a chi‐square test. The interaction was removed from models in which it was not significant. Post hoc pairwise comparison was performed using the *emmeans* function to test for differences between generations and rearing host. For all analyses with *emmeans*, we used the default adjustment from the *emmeans* package for multiple testing, which is false discovery rate, a method based on the Bonferroni adjustment (Benjamini & Hochberg, 1995).

#### 2.6.2 Sex ratio

First, we assessed if treatment (rearing host) over time influenced whether at least one female emerged. In arrhenotokous species, like most parasitoids (Heimpel & De Boer, 2008) unmated females produce male offspring (coded as 0s) and mated females produce both male and female offspring (coded as 1 when at least one female emerged from a trial). We were interested in how selection on *D. suzukii* influenced the sex ratio over 0, 3, and 10 generations. For those trials with successful mating, we then asked how the proportion of emerging females (values bounded between 0 and 1) changed over time in the different treatments. We used generalized linear mixed models with a binomial distribution for both analyses. The fixed terms in both models were the generation (0, 3, or 10), treatment (rearing host), and the replicate population nested in treatment was included as a random effect. We tested for interaction between generation and rearing host using the *anova* function with a Chi‐square test. The interaction was removed from models in which it was not significant. Post hoc pairwise comparisons using the *emmeans* function were used to compare differences between generation and rearing host.

#### 2.6.3 Development time

A linear mixed model was used to compare the development times of the parasitoids in the selection and controls lines in generation 10. The fixed terms in the model were treatment (rearing host) and the sex of the emerging parasitoid. The random effects were the replicate population nested within treatment and the individual female parent.

#### 2.6.4 Body size

A linear mixed model was used to compare the body size (mass) of the parasitoids after 10 generations of rearing on either *D. suzukii* or *D. melanogaster*. Treatment (rearing host) was the fixed term, and the random effect was the replicate population nested within treatment.

#### 2.6.5 Host preference

To test whether host preference evolved, we compared host choices of parasitoids from the different treatments in generations 0, 3, and 10. Preference was defined as the difference between the number of parasitoids emerging from *D. melanogaster* and *D. suzukii*. This creates a metric where zero indicates there was an equal number of parasitoids that emerged from *D. melanogaster* and *D. suzukii*. Numbers greater than zero indicate preference for *D. melanogaster* and the scale indicates the level of preference. Numbers less than zero indicate preference for *D. suzukii* and the degree to which the parasitoids preferred *D. suzukii*. For all analyses, trials in which no parasitoids emerged from both host species were excluded. If some parasitoids emerged from *D. melanogaster* but none from *D. suzukii* or vice versa, those were included.

Given that in generation 0 the selection treatments had not been implemented yet to assess evolution of host preference over time (in generations 0, 3, and 10), the control and selection lines were analyzed separately. The fixed effect in both models was generation, and the replicate population was included as a random effect.

## 3 RESULTS

### 3.1 Developmental success

**FIGURE 1:**
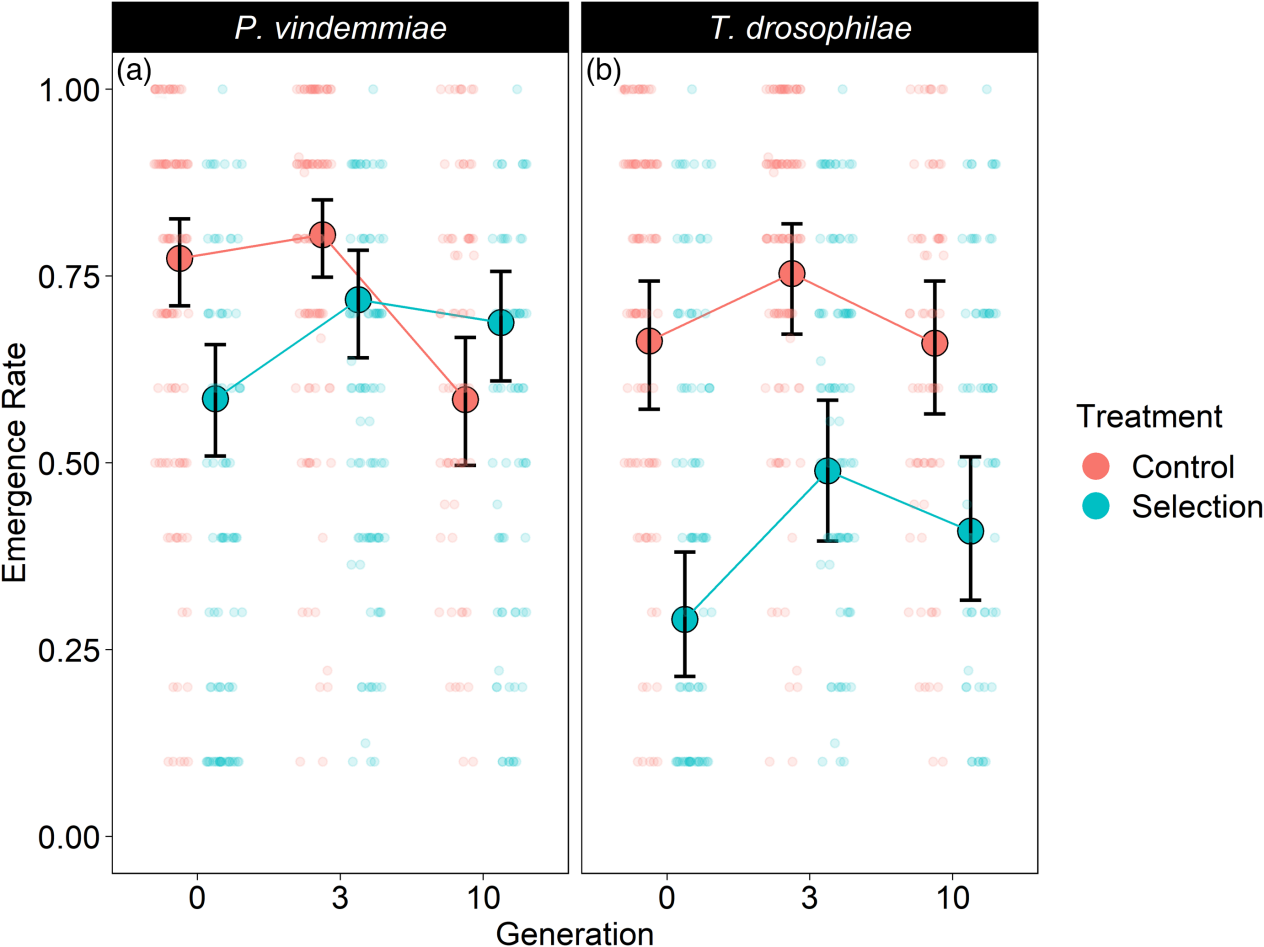
The proportion of either *Drosophila suzukii* (selection treatment) or *Drosophila melanogaster* (control treatment) pupae that had successful parasitoid emergence of either *Pachycrepoideus vindemmiae* (a) or *Trichopria drosophilae* (b) during laboratory selection over 10 generations. Means ± 95% confidence intervals are shown for three replicated populations for each treatment in each generation. Transparent dots display the raw data and are jittered to reduce the overlap. Both for *P. vindemmiae* and *T. drosophilae*, emergence rates on *D. suzukii* increased between generation 0 and 3, and generation 0 and 10, but not between generation 3 and 10. The emergence rate of the control populations of *P. vindemmiae* on *D. melanogaster* decreased between generation 3 and 10, and generation 0 and 10. The emergence rate of the control populations of *T. drosophilae* increased between generations 0 and 3, but then decreased between generations 3 and 10 resulting in no change overall from generation 1 to 10 (see Section 3 for details).

**FIGURE 2:**
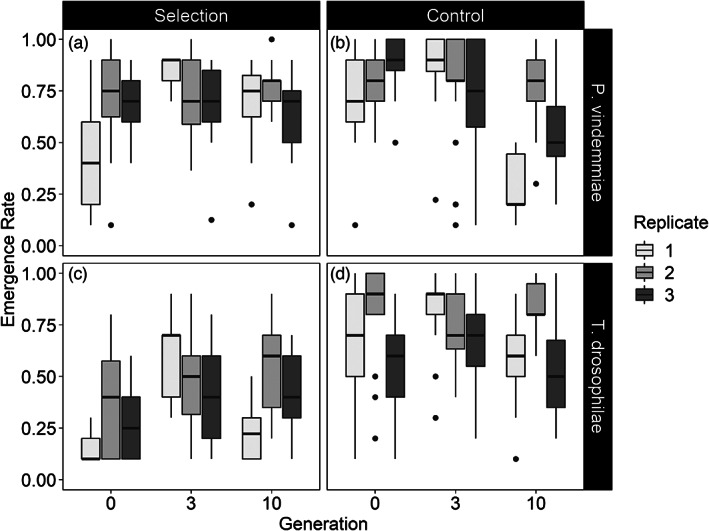
The proportion of either *Drosophila suzukii* (selection treatment) or *Drosophila melanogaster* (control treatment) pupae that had successful parasitoid emergence of either *Pachycrepoideus vindemmiae* (a and b) or *Trichopria drosophilae* (c and d) during experimental evolution over 10 generations. Replicates are shown in different shades of grey. Dots indicate outlier observations, the horizontal line indicates the median with the box representing the interquartile range, and vertical lines are 1.5 times the interquartile range.

The probability that at least one *P. vindemmiae* adult emerged was not affected either by generation or treatment (all *p* > 0.06). Over the 10 generations of the experiment, the number of emerging wasps from each vial increased more for the selection lines than the control lines (Figure S1). The emergence rates of *P. vindemmiae* adults in the selection and control lines showed different trajectories over time (generation × treatment interaction: *z* = 5.41, *p* < 0.001) (Figure 1a). For the selection lines, emergence rates on *D. suzukii* significantly increased between generations 0 (58.6 [50.9%, 65.8%]; mean [95% confidence interval], *N* = 41) and 3 (71.8 [64.1%, 78.5%], *N* = 28) (pairwise comparison: *p* = 0.0179), and from generation 0 to 10 (68.8 [61.0%, 75.6%], *N* = 29) (pairwise comparison: *p* < 0.001). The variation in the performance of the replicate populations was reduced as selection proceeded (Figure 2a). For the control lines, emergence rates on *D. melanogaster* were similar between generation 0 (77.4 [71.0%, 82.7%], *N* = 40) and 3 (80.5 [74.8%, 85.2%], *N* = 48) (*z* = −1.88, *p* = 0.41), and then decreased significantly by generation 10 (58.5 [49.7%, 66.8%], *N* = 23) compared to both generation 0 (pairwise comparison: *p* = 0.005) and generation 3 (pairwise comparison: *p* < 0.001). This decline was driven by one of the replicate populations (Figure 2b).

For *T. drosophilae*, the probability of at least one offspring emerging differed between treatments and over time (treatment × generation interaction: *z* = 2.697, *p* = 0.007). In the selection lines, emergence probability on *D. suzukii* increased from generation 0 (68.5 [44.4%, 85.6%], *N* = 66) to generation 3 (97.6 [88.3%, 99.5%], *N* = 61) (pairwise comparison: *p* = 0.003), but by generation 10, selection lines were not any more likely to produce offspring (89.8 [71.5%, 96.9%], *N* = 90) compared either to generation 0 (pairwise comparison: *p* = 0.101) or to generation 3 (pairwise comparison: *p* = 0.481). In the control lines, there was no change over time in the probability that the females would produce at least one offspring on *D. melanogaster* (all *p* > 0.94). The number of emerging wasps from each vial increased more for the selection lines than the control lines through the 10 generations (Figure S2). Emergence rates of *T. drosophilae* were higher on *D. melanogaster* compared to *D. suzukii* (treatment: *z* = −5.48, *p* < 0.0001) (Figure 1a). Changes in emergence rates between the generations varied for the selection and control lines (generation 3 × treatment interaction: *z* = 2.14, *p* = 0.032; generation 10 × treatment interaction: *z* = 2.66, *p* = 0.008). The pattern of emergence rates for the selection lines of *T. drosophilae* were similar to those of *P. vindemmiae* on *D. suzukii* showing an increase between generation 0 (29.1 [21.4%, 38.1%], *N* = 42) and generation 3 (48.9 [39.5%, 58.4%], *N* = 59) (pairwise comparison: *p* < 0.0001), and between generation 0 and generation 10 (40.9 [31.6%, 50.8%], *N* = 39) (pairwise comparison: *p* = 0.006), but not between generations 3 and 10 (pairwise comparison: *p* = 0.136). In the control lines, emergence rates on *D. melanogaster* first increased between generation 0 (66.3 [57.2%, 74.4%], *N* = 58) and generation 3 (75.3 [67.2%, 82.0%], *N* = 54) (pairwise comparison: *p* = 0.014) and then decreased between generation 3 and generation 10 (66.0 [56.5%, 74.3%], *N* = 44) (pairwise comparison: *p* = 0.0197). This resulted in no change overall in emergence rates from generation 0 to 10 (pairwise comparison: *p* = 1.000). Variability among the three replicate populations appears to be higher for both the control and selection lines in *T. drosophilae* compared to *P. vindemmiae* (Figure 2c,d).

### 3.2 Sex ratio

**FIGURE 3:**
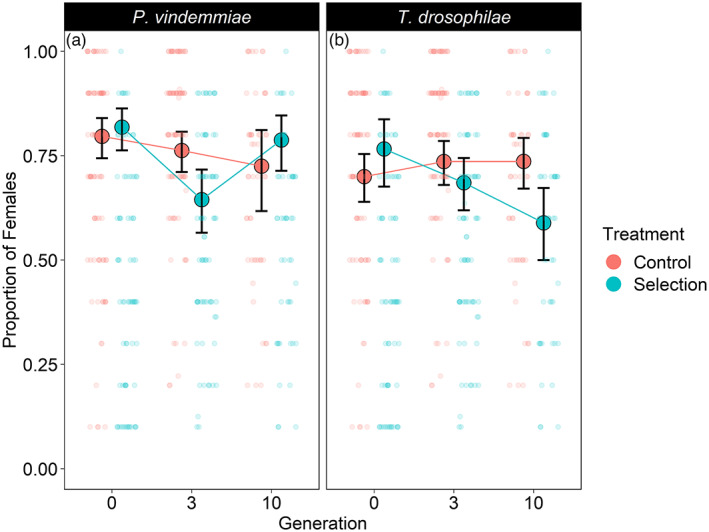
The proportion of *Pachycrepoideus vindemmiae* and *Trichopria drosophilae* females emerging from *Drosophila suzukii* (selection treatment) or *Drosophila melanogaster* (control treatment) during laboratory selection over 10 generations. The mean ± 95% confidence intervals of three replicated populations are shown for each generation and treatment. Transparent dots display the raw data and are jittered to reduce the overlap. For *P. vindemmiae*, the proportion of females emerging on *D. suzukii* decreased between generations 0 and 3 but then rebounded in generation 10 (left panel). For *T. drosophilae*, the proportion of females emerging on *D. suzukii* decreased from generations 0 to 10 (right panel). For both *P. vindemmiae* and *T. drosophilae*, the proportion of females emerging on *D. melanogaster* did not change throughout the experiment (see Section 3 for details).

For *P. vindemmiae*, the probability of at least one female emerging decreased from generation 0 to generation 10 (*z* = −3.45, *p* < 0.0001) on both *D. suzukii* (pairwise comparison: *p* = 0.008) and on *D. melanogaster* (pairwise comparison: *p* = 0.008) (114 trials with no emergent parasitoids excluded). There was a significant interaction between generation and rearing host influencing the sex ratio for *P. vindemmiae* (*z* = −2.26, *p* = 0.02) that stemmed from the selection treatment showing a decrease in the proportion of females from generation 0 (81.9 [76.3%, 86.4%], *N* = 38) to generation 3 (64.5 [56.6%, 71.7%], *N* = 21) (pairwise comparison: *p* = 0.002) (Figure 3a). However, by generation 10, the sex ratio on *D. suzukii* rebounded (78.8 [71.4%, 84.7%], *N* = 21) and was similar to generation 0 (pairwise comparison: *p* = 0.978). For the control lines, there was no significant change in sex ratio for any generation (*p* > 0.76 for all pairwise comparisons of generations).

For *T. drosophilae*, the probability of at least one female emerging was not different from the populations at generation 0 (generation 3: *z* = 0.81, *p* = 0.417; generation 10: *z* = −0.9, *p* = 0.367). It was more likely that at least one female would emerge when reared on *D. melanogaster* compared to *D. suzukii* (treatment: *z* = −0.78, *p* = 0.044) (55 trials with no emergent parasitoids excluded). There was a significant interaction between generation and rearing host influencing the sex ratio (*z* = −3.05, *p* = 0.002) (Figure 3b) that stemmed from a decline in the proportion of females in the selection lines from generation 0 (76.6 [67.6%, 83.7%], *N* = 36) to generation 10 (58.9 [50.0%, 67.3%], *N* = 31) (pairwise comparison: *z* = 3.02, *p* = 0.03). For the control lines, there was no significant change in the sex ratio between any generations (*p* > 0.89 for all generations).

### 3.3 Development time

There was no difference in development time between the selection and the control lines for either *P. vindemmiae* (*t* = −0.87, *p* = 0.43) or *T. drosophilae* (*t* = 0.55, *p* = 0.61). As expected, males had a significantly shorter development time than the females in both *P. vindemmiae* (*t* = −5.14, *p* < 0.001) and *T. drosophilae* (*t* = −7.07, *p* < 0.001). For *P. vindemmiae*, the average development time for males was 15.3 [13.9, 16.8] days (*N* = 74) in the control lines and 14.7 [13.3, 16.1] days (*N* = 88) in the selection lines. For *P. vindemmiae* females, the average development time was 16.1 [14.7, 17.5] days (*N* = 51) in the control lines and 15.5 [14.0, 16.9] days (*N* = 115) in the selection lines. For *T. drosophilae*, the average development time for males was 18.5 [17.4, 19.5] days (*N* = 92) in the control lines and 18.8 [17.7, 19.8] days (*N* = 72) in the selection lines. For *T. drosophilae* females, the average was 19.6 [18.5, 20.7] days (*N* = 182) in the control lines and 19.9 [18.8, 21.0] days (*N* = 86) in the selection lines.

### 3.4 Body size

There was no difference in body size between the selection lines and the control lines for either *P. vindemmiae* (*t* = 1.66, *p* = 0.18) or *T. drosophilae* (*t* = 0.24, *p* = 0.82) in generation 10. For *P. vindemmiae*, the mean female mass in the control lines was 0.158 [0.113, 0.202] mg (*N* = 61) and 0.195 [0.150, 0.240] mg (*N* = 70) in the selection lines. For *T. drosophilae*, the average mass of the females was 0.119 [0.77, 0.160] mg in the control (*N* = 87) and 0.124 [0.830, 0.165] in the selection lines (*N* = 81).

### 3.5 Host preference

**FIGURE 4:**
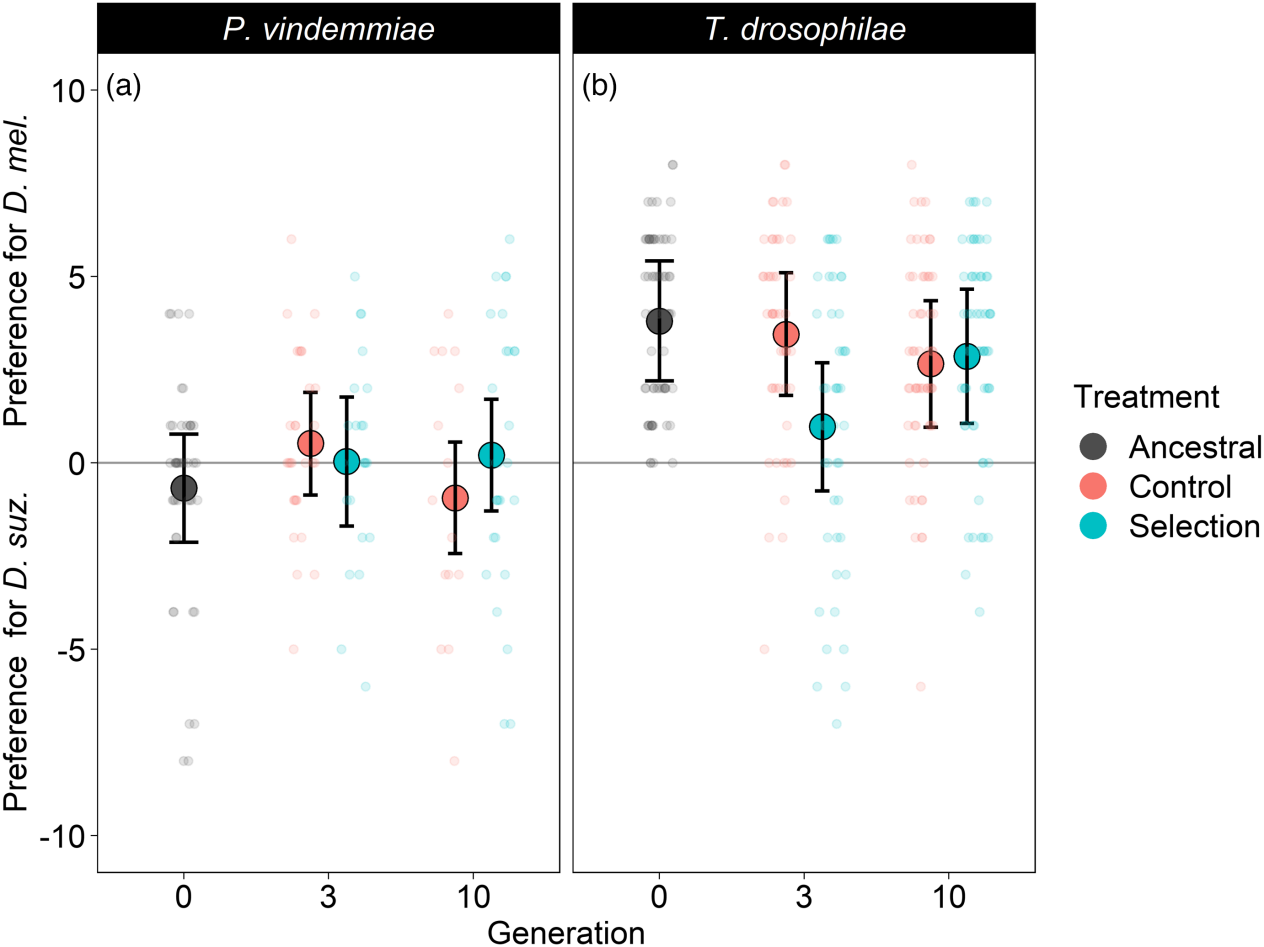
The preference of *Pachycrepoideus vindemmiae* and *Trichopria drosophilae* parasitoids reared on *Drosophila suzukii* (selection) or reared on *Drosophila melanogaster* (control) in generations 0, 3, and 10. Preference was defined as the number of parasitoids emerging from *D. melanogaster* minus the number of parasitoids emerging from *D. suzukii*. The mean ± 95% confidence intervals of three replicate populations are shown for each generation and treatment. Transparent dots display the raw data and are jittered to reduce the overlap. For *P. vindemmiae*, preference did not change throughout the experiment in either the control or the selection treatments. For *T. drosophilae*, preference did not change in the control treatment. For the selection treatment, *T. drosophilae* increased preference towards *D. suzukii* between generations 0 and 3 but moved back towards increased preference for *D. melanogaster* in generation 10 (see Section 3 for details).

For *P. vindemmiae*, there was no change of preference in the selection lines (*p* > 0.32 for all generations) or the control lines (*p* > 0.14 for all generations) (Figure 4a). In the *T. drosophilae* selection lines, females increased their preference towards *D. suzukii* from generation 0 to generation 3 (*z* = −4.51, *p* < 0.001) (Figure 4b). However, this intermittent increase in preference towards the novel host was not evident by generation 10 (*z* = −1.64, *p* = 0.10). In the control lines, preference did not change between generations 0 and 3 (*z* = −0.62, *p* = 0.53). In generation 10, the control lines preferred *D. melanogaster* slightly less compared to the original population (*z* = −2.04, *p* = 0.04).

## 4 DISCUSSION

Previously, we found that two pupal parasitoids, *P. vindemmiae* and *T. drosophilae*, adapted rapidly to a novel host, *D. suzukii*, substantially increasing their developmental success in just three generations of selection (Jarrett et al., 2022). Here, we show that additional seven generations of selection do not result in further improvement compared to that achieved by generation three. Adaptation was not accompanied by increased preference towards the novel host and there were no fitness trade‐offs in development time or body size associated with increased developmental success on *D. suzukii*. Initially, the sex ratio became less female‐biased for both parasitoid species undergoing selection, but it recovered in *P. vindemmiae* over time.

The speed at which parasitoids respond to selection on a novel host can be rapid but there can also be limits to the level of adaptation achieved. For example, parasitism by *Aphelinus rhamni* (Hopper and Woolley) (Hymenoptera: Aphelinidae) on the novel aphid host, *Rhopalosiphum padi* (L.) (Hemiptera: Aphididae) increased significantly after two generations of selection but further improvement was not observed in generation three (Hopper et al., 2021). Despite the rapid response to selection, *A. rhamni* parasitism on the novel host remained well below parasitism rates on its original host of *Aphis glcyines* (Matsumura) (Hemiptera: Aphididae) even after 150 generations of selection (Hopper et al., 2021), which could be partly due to its initial restricted host range. Similarly, we found that the response to selection of both *P. vindemmiae* and *T. drosophilae* was initially rapid but developmental success did not continue to increase after the third generation of selection for either species. *Trichopria drosophilae*, whose host range is more restricted than of *P. vindemmiae*, had lower initial developmental success (29%) on the novel *D. suzukii* than *P. vindemmiae* (58%), which is more of a generalist that can parasitize several Dipteran species (Wang & Messing, 2004). The rate of improvement was greater for *T. drosophilae* as they increased their developmental success from 29% to 41%–49% by generations three and 10 but that was still under the 66%–75% emergence rates recorded on *D. melanogaster*. On the other hand, the generalist *P. vindemmiae* showed less quantitative improvement; 68%–72% emergence rates after selection compared to the 58% initial developmental success. However, this increase was enough to reach similar emergence rates on the novel host than on the original host (58%–81%). In other words, the performance gap between the original and novel host was smaller for the generalist than the more specialized parasitoid, which the generalist parasitoid successfully closed whereas the specialist did not reach similar developmental success on the novel host. These results are not surprising given the differences in the life history strategies of the two parasitoids. While both species are pupal parasitoids, *P. vindemmiae* is an ectoparasitoid, and *T. drosophilae* is an endoparasitoid (Wang et al., 2020). Ectoparasitoids lay their eggs outside the host tissue, between the puparium case and the pupae, and thus do not have to contend with an immune response from the host, unlike endoparasitoids (Godfray & Godfray, 1994; Wang et al., 2020). Therefore, the adaptations of ecto‐ and endo‐parasitoids to novel or sub‐optimal host species likely involve different mechanisms and are expected to show differences in the rate and upper limit of adaptation.

In addition to the life history strategies used by parasitoids, the genetic variation within populations is another important component that influences the response to selection (Barrett & Schluter, 2008). Reduced genetic variance could have constrained adaptation in the aphid endoparasitoid *A. rhamni* and may have led to the rapid leveling of fitness after just two generations (Hopper et al., 2021). In a study with *A. ervi* where parasitism rates of selected populations on the novel foxglove aphids kept increasing for up to 40 generations, the parasitoid populations were started with over 2700 individuals that likely captured most of the available genetic variation locally (Henry et al., 2008). On the other hand, the larval endoparasitoid *L. heterotoma* could not evolve to overcome the strong immune response of *D. suzukii* larvae over seven generations of selection despite using a diverse population that was derived by mixing seven distinct strains collected across Europe (Kruitwagen et al., 2021). We started both parasitoid populations with relatively few individuals (2–30 individuals) (Jarrett et al., 2022), which likely resulted in reduced genetic variation that could have limited adaptive ability and might explain why no further improvement was seen after generation three. The relatively low initial genetic variation, the fluctuating population sizes, and the multi‐generation laboratory rearing could also have resulted in genetic drift, which may explain some of the variation seen among the replicate populations. In general, too little is known about the genetics of host adaptations in parasitoids (Hopper et al., 2019, 2021) to evaluate how genetic constraints and other genetic and demographic processes may interact to influence the outcome of selection. Even though we demonstrated that adaptation is possible using few founders, clearly, we would recommend starting with higher founding population sizes when implementing laboratory selection.

We know that adaptation to novel hosts can be accompanied by correlated changes in behavior and in various fitness traits (Dion et al., 2011; Henry et al., 2008; Jones et al., 2015). For example, egg laying in a sub‐optimal host can result in smaller body size, longer development time, and changes in the sex ratio of the brood (Charnov, 2020; Dion et al., 2011; Godfray & Godfray, 1994; West & Sheldon, 2002). Body size is important in female fitness as it is correlated with greater fecundity and longevity (Beukeboom, 2018; Sagarra et al., 2001). Longer development time can be maladaptive as it increases the chances of predation (Doyon & Boivin, 2005). In parasitoids, female‐biased sex ratios are favored to reduce mate competition and increase population growth and it is well‐documented that more males are produced in poor‐quality hosts (Abe & Kamimura, 2012; Godfray & Godfray, 1994; Hamilton, 1967; Hardy, 1994; West et al., 2005). While we did not find any negative fitness correlations in terms of body size or developmental time, between generations 0 and 3 sex ratio of both parasitoid species became less female‐biased on the novel host. This could have been due either to the female parasitoids laying fewer fertilized eggs or because female progeny had lower survival in the novel host. The sex ratio of *P. vindemmiae* rebounded by generation 10, but *T. drosophilae* continued to produce more males on the novel compared to the original host. The differences in the responses of the two parasitoids could be related to their host range and the initial performance on the novel host. As mentioned previously, the generalist *P. vindemmiae* successfully closed the initial performance gap between the novel and original host. Thus, it is not surprising that by reaching similar developmental success on the novel host it was also able to produce broods with similarly female‐biased sex ratios as on the original host. Since *T. drosophilae* developmental success on *D. suzukii* has not reached the levels seen on *D. melanogaster*, the novel host can still be considered sub‐optimal, which is reflected in the higher male production. Thus, it may be more likely that the sex ratio in this case was mediated by the females assessing the suitability of the host and deciding to lay fewer fertilized eggs in those deemed sub‐optimal. While a shift in sex ratio towards more males could reduce parasitism in the field, overall, sex ratios of both parasitoids remained female‐biased, suggesting that the selected populations could maintain positive growth rates over time.

For host range expansion to occur, where the novel host is consistently attacked in the field, both behavioral and physiological adaptations are necessary (Agosta, 2006; Antolin et al., 2006; Henry et al., 2008, 2010). To ensure continued attack, changes in host fidelity or preference towards the novel host should accompany the increased developmental success. Parasitoids often prefer the host in which they developed (Daza‐Bustamante et al., 2002; Pennacchio et al., 1994), a process that can promote specialization and further adaptation to a novel host (Fry, 1996; Henry et al., 2008). For example, the host fidelity of *A. ervi* increased on the novel foxglove aphid after a single generation of rearing, which was deemed a plastic response as it did not further increase as selection progressed even as virulence kept increasing (Henry et al., 2008). In our experiments, *P. vindemmiae* showed no preference for either host species throughout the 10 generations of selection. Such a lack of preference could make it easier for generalists such as *P. vindemmiae* to adapt to novel hosts as they are less likely to be deterred by low initial survival rates. However, it also means that selected populations of *P. vindemmiae* may be most useful in environments where the density of *D. suzukii* is much higher than the density of other host species since it may attack the host with which it comes in contact most (Kaçar et al., 2017). In locations where multiple hosts are available, the disruptive selection to maintain the ability to attack multiple species would interfere with the increased developmental success of populations selected in the laboratory and then released into outdoor settings. *Trichopria drosophilae* showed preference towards the original host throughout the experiment with a slight shift towards increased preference of the novel host by generation 3, which was not observed by generation 10. For specialists such as *T. drosophilae*, it may be more difficult for preference to shift (Poisot et al., 2011). Host selection behavior has been shown to correlate with survival probability (Kraaijeveld et al., 1995). Hence, the lower survival rates on *D. suzukii* relative to that on *D. melanogaster*, even after 10 generations of selection, could have prevented an increase in preference for *T. drosophilae*. Therefore, *T. drosophilae*, which has a clear preference for its co‐evolved host, may be unlikely to attack the novel host, *D. suzukii*, in the presence of native Drosophilid hosts.

Both *P. vindemmiae* and *T. drosophilae* have already been used in augmentative releases against *D. suzukii*. Releases of *T. drosophilae* are reported to reduce *D. suzukii* populations by up to 50% (Gonzalez‐Cabrera et al., 2021; Stacconi et al., 2015). Given its narrower host range, *T. drosophilae* may be the preferred parasitoid to use in augmentative releases even if it may have lower fitness on *D. suzukii* than *P. vindemmiae*, to reduce any possible nontarget effects on native Diptera species. So far, augmentative releases of *P. vindemmiae* have had less success, but this may be partly due to the small release sizes (Hogg et al., 2022). Additionally, the studies releasing *T. drosophilae* reared the parasitoids on *D. suzukii* before release while the study by Hogg et al. (2022) reared *P. vindemmiae* on *D. melanogaster* prior to release. Our study suggests that rearing native parasitoids on the invasive target host for at least three generations could make augmentative releases more effective but rearing beyond that is unlikely to yield additional benefits in terms of increasing levels of adaptation.

This study is an important step towards better understanding how laboratory selection of native parasitoids may be used as an alternative or complementary approach to classical biological control to control invasive insect pests. In addition, it contributes to our limited knowledge of how insect parasitoid host ranges may evolve as it is the first to compare responses of a generalist and a more specialized parasitoid to selection on a novel host species by also assessing correlated responses. It is yet to be determined how adaptation induced in the laboratory will translate to field conditions and how correlated fitness and behavioral responses may impact the long‐term evolutionary trajectories of the selected populations.

## CONFLICT OF INTEREST

The authors declare no conflict of interest.

## Supporting information


Appendix S1

## Data Availability

Data for this study are available at: https://doi.org/10.5061/dryad.wwpzgmsns.
