# Supplementary material for: Limited gains in native parasitoid performance on an invasive host beyond three generations of selection
Source: Evol Appl. 2022 Nov 2;15(12):2113–24. doi: 10.1111/eva.13504 (PMC9753813; doi:10.1111/eva.13504)
Supplement: Supplementary file 1 — Appendix S1 [file EVA-15-2113-s001.docx]

**Supplementary Materials**

Generation analysis summary

**Methods**

We counted the number of emerging wasps from each vial for every generation. Vials were standardised by the number of flies put in to lay eggs, and the number of parasitoids put in to parasitise the pupae. Numbers, however, could vary due to sex ratios and stochasticity, and thus we caution interpretation of these data, which are solely presented to give a broad overview of the experiment. We used a simple generalised linear mixed model for each parasitoid species, where generation (as a continuous variable to get a general pattern over time) was included in a model with its interaction with fly species (*D. melanogaster* or *D. suzukii*). The population replicate was included as a random term. We used a negative binomial distribution for the data in the R package brms (Bürkner 2017). We compared the model with the interaction against the model excluding the interaction to assess whether the slopes for *D. melanogaster* and *D. suzukii* were different using WAIC (Vehtari et al. 2017).

**Results**

We found support that the slopes for the populations evolving on *D. melanogaster* were shallower than the slopes for the populations evolving on *D. suzukii* for both *P. vindemmiae* (Fig. S1) and *T. drosophilae* (Fig. S2), though the support for this interaction is more substantial for *T. drosophilae*. This indicates that the numbers of parasitoids emerging from each vial increased more for the *D. suzukii*-evolving populations than the *D. melanogaster*-evolving populations over the course of the ten-generation experiment. For *P. vindemmiae*, the interaction model had a slightly lower WAIC (ΔWAIC = 0.80) and the majority of the model weight (weight = 0.80). The *D. melanogaster*-evolving populations had a weakly positive slope (β = 0.02 ± 0.01) whilst the *D. suzukii*-evolving populations had a positive slope (β = 0.04 ± 0.01). For *T. drosophilae*, the model with the interaction had a much lower WAIC value (ΔWAIC = 23.88) and almost all the model weight (weight = 0.99). The *D. melanogaster*-evolving populations had a slightly negative slope (β = –0.03 ± 0.01) whilst the *D. suzukii*-evolving populations had a positive slope (β = 0.06 ± 0.02).


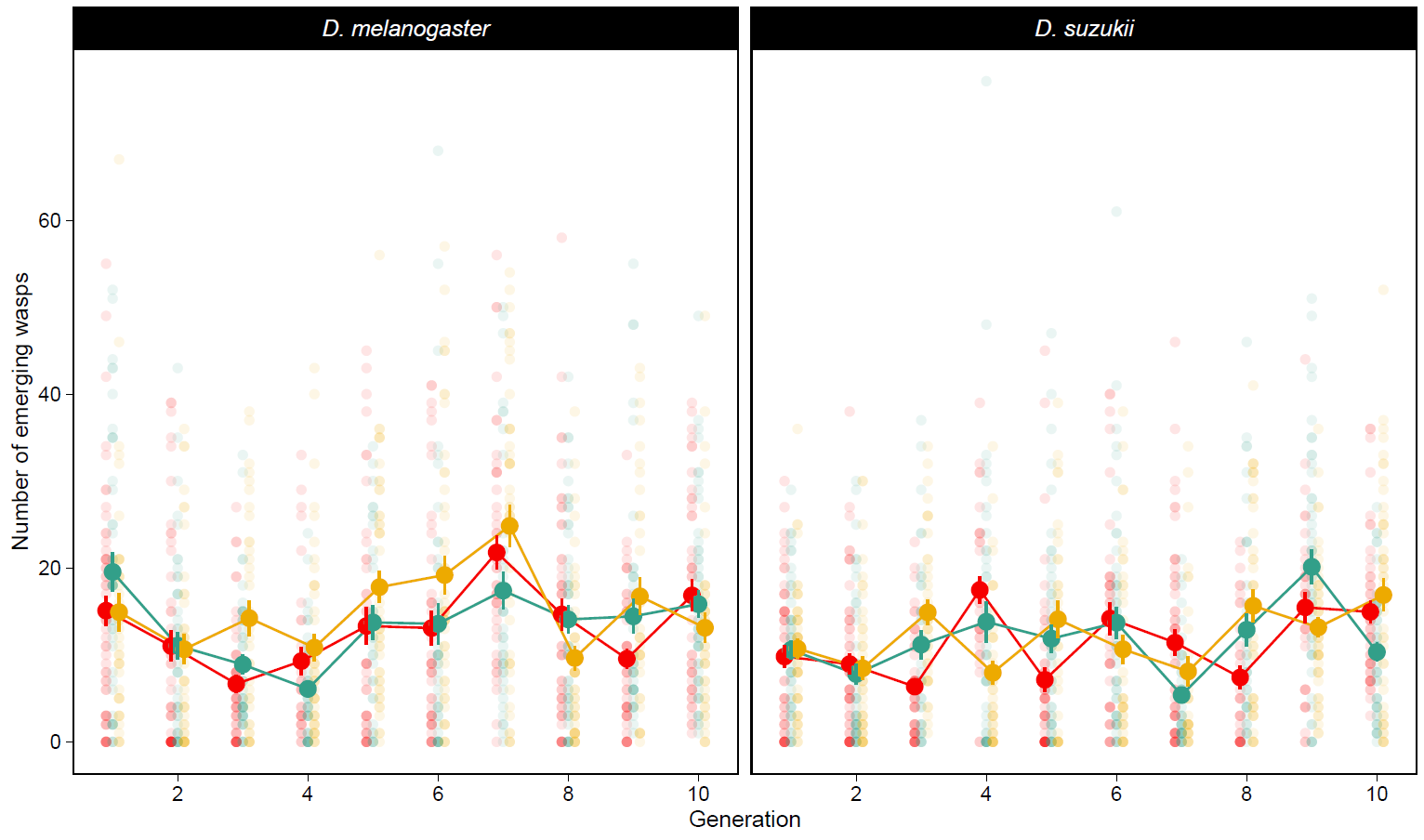


**Fig. S1**. The number of emerging *Pachycrepoideus vindemmiae* individuals from vials of *Drosophila melanogaster* (left panel) and *D. suzukii* (right panel) over the course of the ten generations of evolving on each respective host. The colours represent the three replicate populations per treatment.


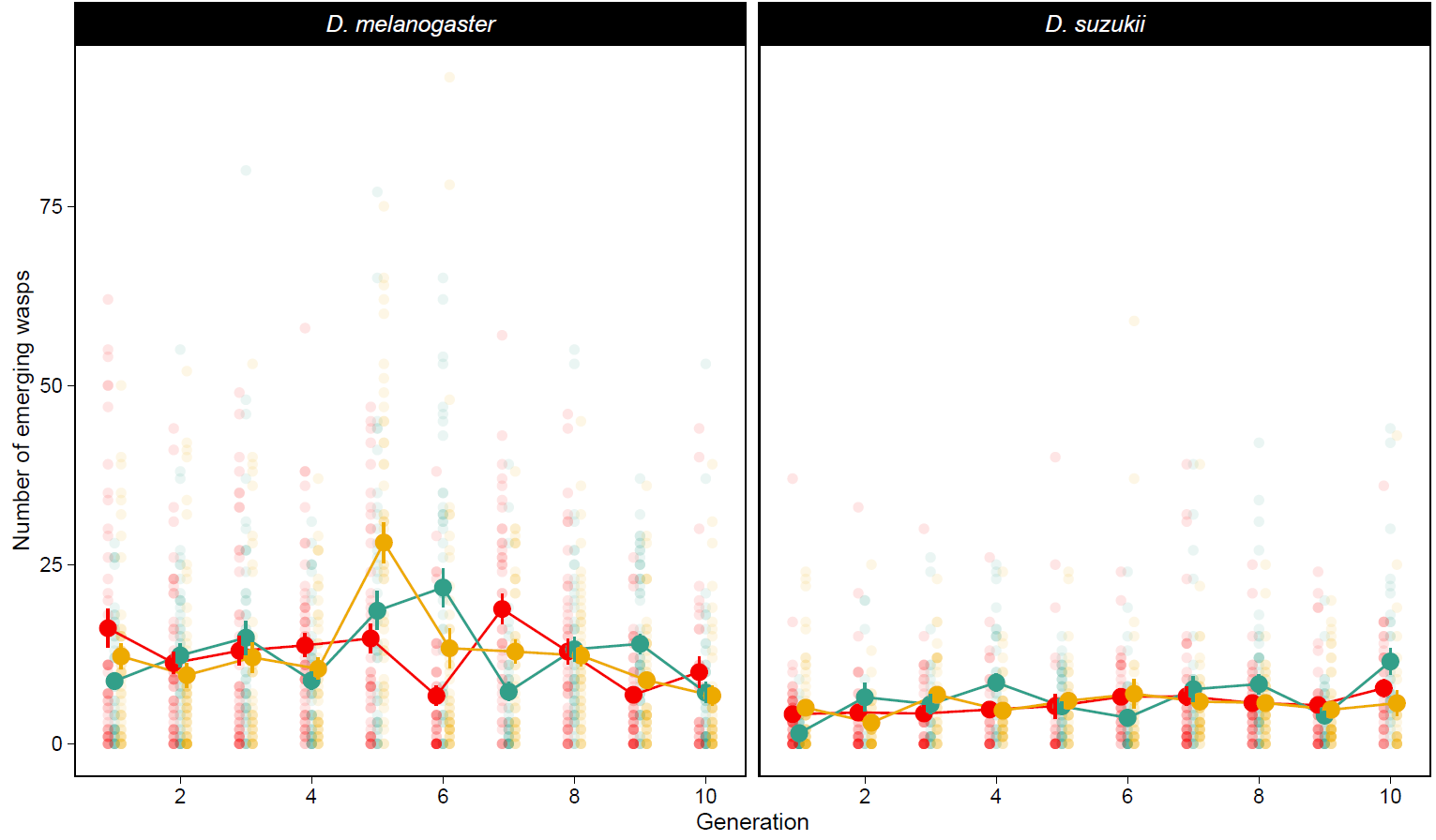


**Fig. S2**. The number of emerging *Trichopria drosophilae* individuals from vials of *Drosophila melanogaster* (left panel) and *D. suzukii* (right panel) over the course of the ten generations of evolving on each respective host. The colours represent the three replicate populations per treatment.

**References**

Bürkner P-C (2017) brms: An R package for Bayesian multilevel models using Stan. Journal of Statistical Software 80, 1–28

Vehtari A, Gelman A, and Gabry J (2017) Practical Bayesian model evolutionary using leave-one-out cross-validation and WAIC. Statistics and Computing 27, 1413–1432
